# Preventing Dengue Epidemics during the COVID-19 Pandemic

**DOI:** 10.4269/ajtmh.20-0480

**Published:** 2020-06-15

**Authors:** Annelies Wilder-Smith, Hasitha Tissera, Eng Eong Ooi, Josefina Coloma, Thomas W. Scott, Duane J. Gubler

**Affiliations:** 1 Heidelberg Institute of Global Health, University of Heidelberg, Heidelberg, Germany;; 2 Ministry of Health, Colombo, Sri Lanka;; 3 Duke-NUS, Singapore;; 4 University of California, Berkeley, Berkeley, California;; 5 University of California, Davis, Davis, California

The COVID-19 pandemic is placing immense pressure on healthcare and public health systems worldwide. In addition to the COVID-19–related death toll and increased demand on intensive care, the pandemic has major repercussions on health outcomes of other diseases at times when resources and personnel are diverted to COVID-19. This challenge is of particular concern for low- and middle-income countries. WHO has emphasized the need to sustain efforts to prevent, detect, and treat vector-borne diseases, such as malaria, during this pandemic.^1^ A similar approach should be adopted for the prevention and control of dengue and other arboviral diseases.

About 100 million dengue cases occur every year, with the highest burden in Southeast Asia followed by Latin America.^2^ Epidemic transmission of both dengue and COVID-19 is driven by population densities.^2,3^ Thus, the impact of combined dengue and COVID-19 epidemics could have potentially devastating consequences in tropical and subtropical cities. Manifestations of this double burden are seen in Guayaquil, Ecuador, and Iquitos, Peru, where an enormous dengue season coincided with the spread of SARS-CoV-2, and extreme excess mortality cannot be attributed only to COVID-19.^4^ The dengue incidence rate was 12.86 cases/100,000 inhabitants in the region for the ongoing year, including 27 deaths, 12,891 cases confirmed by laboratory, and 498 cases classified as severe dengue (0.4%).^4^ Countries such as Bolivia, Honduras, Mexico and Paraguay have reported an increase of double or triple the number of cases of dengue compared with the same period from the previous year,^5^ and the humanitarian and migration crisis in Venezuela has led to a breakdown in public health structures.^6^ Southeast Asia is also battling a surge in dengue cases.

In the early stages, dengue and COVID-19 are difficult to distinguish because they share similar clinical and laboratory features early on. Both often initially present as undifferentiated fever with nonspecific signs and symptoms; shared laboratory parameters include lymphopenia, leukopenia, thrombocytopenia, and elevated transaminases.^7^ Singapore reported on patients being first incorrectly diagnosed with dengue due to a false-positive dengue rapid serological test who were later confirmed to be COVID-19.^7^ Misdiagnosis of COVID-19 as dengue with failure to isolate such patients will trigger outbreaks in healthcare settings and beyond. Failing to recognize dengue and institute timely hydration may lead to preventable dengue-related deaths. High awareness of dengue and application of virological tests to differentiate dengue from COVID-19 is thus a priority in healthcare systems throughout the tropics and subtropics. At the same time, universal personal protective measures need to be applied at all times to prevent inadvertent SARS-CoV-2 transmission in healthcare centers. Although severe outcomes for COVID-19 appear to be predominantly in older persons,^8^ whereas dengue affects more children and young adults in most dengue-endemic countries,^2^ dengue has also emerged to be a major cause of morbidity and mortality in the elderly in places such as Singapore and Taiwan. A detailed and strict triage algorithm to differentiate the diseases should be followed. Coinfection with both viruses is possible and needs to be excluded.

Although public health staff in many countries are currently diverted to COVID-19 response, it is of utmost importance to maintain and enhance mosquito control measures during the pandemic. For countries where governments have introduced lockdowns, special permissions should be sought to continue vector control strategies. Vector control teams targeting mosquito breeding sites should comply with social distancing measures and wear personal protective equipment (PPE) if entering houses for indoor residual insecticide spraying. Wearing PPE in hot and humid climates is cumbersome. The international community needs to develop new PPE approaches that are more amenable to such climates.

Teams conducting indoor residual spraying at the household level could combine this activity with active case detection for COVID-19. Health messaging to enhance compliance to COVID-19 public health measures should be combined with messaging to enhance household and community participation in vector control measures.

At a time of lockdowns when people are told to stay at home, community participation in mosquito control activities should be strengthened. Dengue transmission in and around homes is considered a driving factor for dengue outbreaks. Residents should be reminded of the mosquito life cycle, including the aquatic phase of 6–10 days, and encouraged to work together in and around their homes to remove stagnant water, reduce solid waste and plastics that fill with rain, and ensure proper covering of all water storage containers. The intra-domicile application of targeted indoor residual spraying should be selectively directed at *Aedes aegypti* resting places, such as under furniture and on dark surfaces. Precautions must be taken not to fumigate drinking water storage tanks. Tackling breeding sites at homes could become a weekly family activity that should be maintained even after lockdowns are lifted. Vulnerable members within households such as the elderly, pregnant women, infants, and those with underlying medical conditions should be encouraged to use insect repellents to protect themselves against mosquito bites. New innovative strategies could be developed; for example, to combine hand sanitizers against COVID-19 with insect repellents against arboviral diseases vectors.

Studies have shown that buildings and sites beyond the household level can be at increased risk for breeding and proliferation of mosquitoes if no one takes ownership for vector control at those sites.^9^ This is particularly true for schools, cemeteries, and construction sites.^9,10^ Many lockdowns include school closures. It is important to maintain vector control measures at such schools even during closures to keep breeding sites at bay to prevent dengue outbreaks when children return to school after the lockdown. Regular cleaning and fumigation need to be maintained at construction sites and cemeteries even when regular work has been discontinued because of lockdown.

In summary, a resurgence of dengue and other arboviral diseases is a real threat during the COVID-19 pandemic because they add to overwhelming already fragile healthcare systems. Lockdown periods should be leveraged as an opportunity to enhance vector control measures at the household level. At the community level, public health messaging and community engagement should include both diseases. At national and global levels, it is imperative to sustain effective vector control measures.
